# Profiles of *Rho*, *Opn4*, *c-Fos*, and *Birc5* mRNA expression in Wistar rat retinas exposed to white or monochromatic light

**DOI:** 10.3389/fnana.2022.956000

**Published:** 2022-08-18

**Authors:** Natalia Ziółkowska, Bogdan Lewczuk

**Affiliations:** Department of Histology and Embryology, Faculty of Veterinary Medicine, University of Warmia and Mazury in Olsztyn, Olsztyn, Poland

**Keywords:** rhodopsin, melanopsin, photoreceptor damage, monochromatic light, blue light

## Abstract

Despite concern over potential retinal damage linked to exposure to light-emitting-diode (LED) light (particularly blue light), it remains unknown how exposure to low-intensity monochromatic LED light affects the expression of rhodopsin (*Rho*, a photopigment that mediates light-induced retinal degeneration), melanopsin (*Opn4*, a blue-light sensitive photopigment), *c-Fos* (associated with retinal damage/degeneration), and *Birc5* (anti-apoptotic). This study investigated the mRNA expression profiles of these genes under exposure to white and monochromatic light (blue, red, green) in the retinas of albino rats under a cycle of 12 h of light and 12 h of darkness. In each group, 32 Wistar rats were exposed to one type of monochromatic-LED or white-fluorescent light for 7 day (150 lx). Retinal samples were taken for qPCR analysis and light and electron microscopy. Blue and green light exposure markedly decreased expression of *Rho* and *Opn4* mRNA and increased expression of *Birc5* and *c-Fos* mRNA (*P* < 0.05). In retinas from the blue-light group, loss and vesiculation of photoreceptor outer segments were visible, but not in retinas from the red-light and control group. Measurements of the photoreceptor inner and outer segments length revealed, that this length was significantly decreased in the blue- and green-light exposure groups (*P* < 0.02), but not in the red-light exposure group. Increased expression of *Birc5* and decreased expression of *Rho* and *Opn4* after exposure to blue and green light may be early responses that help to reduce light-induced retinal damage.

## Introduction

The retina is the innermost, photosensitive structure of the eye and responsible for visual and non-visual perception, which are made possible by the presence of photosensitive pigments in retinal photoreceptors. Rhodopsin (RHO), which is present in rods, and opsins, which are present in cones, are responsible for scotopic and photopic vision, respectively (Tao et al., 1993). In contrast, in mammals, melanopsin (*OPn4*), is present in intrinsically photosensitive retinal ganglion cells (ipRGCs) and is responsible for circadian photoentrainment and other non-image forming responses, such as the pupillary light reflex and regulation of the sleep-wake cycle (Tosini et al., 2016).

When light hits the retina, it activates retinal photoreceptors, but depending on its wavelength, intensity, and duration, it can also damage this delicate structure (Grimm et al., 2001; Shang et al., 2014, 2017; Jaadane et al., 2015). In general, the retina is most susceptible to damage from light at shorter wavelengths, and the damage caused by light is primarily photochemical in nature (Ham et al., 1976; Gorgels and van Norren, 1995). White light emit-ting diode (LED), which is emitted by smart phones, tablets, and screens, contains a considerable amount of blue light, as it is formed by the emission of red, green, and blue light (Calvo-Sanz and Tapia-Ayuga, 2020). In particular, the negative effects of blue light on the retina have drawn attention due to increasing use of devices emitting blue-enriched light.

In contrast, there is strong evidence indicating that red light (600–1,000 nm) has beneficial effects on different tissues, including the retina. For example, preconditioning of the rat retina with red light protects this tissue from the damaging effects of bright white light exposure (Albarracin and Valter, 2012).

Blue light causes photoreceptor death and retinal gliosis, which leads to retinal de-generation. However, the severity of those changes depends on the intensity and time of exposure (Grimm et al., 2001; Iandiev et al., 2008; Roehlecke et al., 2011; Shang et al., 2014; Geiger et al., 2015; Jaadane et al., 2020).

Rhodopsin is most sensitive to blue light at 470–480 nm and serves as a mediator of light-induced retinal degeneration (Grimm et al., 2001). Rodent studies have shown that blue light causes more damage to the retina than other kinds of light, and exposure to this light causes RHO-mediated photoreceptor apoptosis and necrosis (Grimm et al., 2001). Similarly, the negative effects of light exposure on cones, horizontal cells and the inner retina depend on light absorption by RHO (Samardzija et al., 2019).

In mice that were exposed to white light during photophase, expression of *Rho* mRNA displayed circadian oscillation with maximum values around evening (Von Schantz et al., 1999). In rats kept under a similar light-dark regime, it also showed distinct circadian variation (Kamahis et al., 2005). However, despite the known sensitivity of RHO to blue light, the changes in *Rho* mRNA expression under monochromatic light of different wavelengths have not been investigated.

*OPn4* is expressed in ipRGCs, which take part in regulation of pupillary light reflexes, the sleep-wake cycle, and many others processes (Hannibal et al., 2005, 2013). In albino and pigmented rats, *Opn4* expression displays significant rhythmic changes during the LD cycle, with a peak around dusk and a nadir at dawn (Hannibal et al., 2005, 2013). In isolated retinal explants from chicken embryos, green light promotes circadian expression of *Opn4-1*, *Opn4-2*, clock genes, and retinal melatonin (Bian et al., 2020). However, it is not known how exposure to monochromatic light at other wavelengths affects rhythmic expression of *Opn4*.

Like RHO, *OPn4* is also sensitive to blue light, but it is not clear whether it mediates the damaging effect of light exposure on ipRGCs (Ziółkowska et al., 2022). Although it is known that expression of *OPn4* is reduced and/or downregulated after exposure to damaging (≥ 1,000 lx) white or blue light (Hannibal et al., 2005, 2013; García-Ayuso et al., 2017; Ziółkowska et al., 2022), it has not been investigated whether *Opn4* expression changes after expression to monochromatic light at lower intensity.

The transcriptional factor *c-Fos* is a protein that modulates expression of a variety of genes, including those in the retina (Tao et al., 1993; Kumar et al., 1996) and is a mediator of apoptosis (Smeyne et al., 1993; Preston et al., 1996). It has been suggested that *c-Fos* plays an important role in the regulation of rod-specific gene expression (He et al., 1998). It is expressed in classical and non-classical photoreceptors after light exposure (Nir and Agarwal, 1993; Semo et al., 2003). In the retinal neurons of rats, *c-Fos* mRNA levels are 4-fold higher during scotophase than during photophase (Kamahis et al., 2005). Upregulation of *c-Fos* is associated with photoreceptor apoptosis after white light exposure (Hafezi et al., 1997; Grimm et al., 2000a) and different forms of retinal damage or degeneration (Otori et al., 1997; Oshitari et al., 2014). For example, in damaged retinal ganglion cells (RGCs), *c-Fos* is induced, and it transmits death signals to the mitochondria (Oshitari et al., 2002). Interestingly, an absence of *c-Fos* (i.e., in knock-out mice) prevents light-induced retinal damage. Expression of *c-Fos* in the light-damaged rat retina may be controlled *via* a cone-specific pathway (Harada et al., 1996). In previous studies, changes in *c-Fos* expression were investigated mostly in the context of light-induced retinal damage. However, there is a lack of information about the profile of *c-Fos* mRNA expression under exposure to alternating periods of monochromatic light (blue, red, and green) and darkness.

*BIRC5* is a member of the inhibitor-of-apoptosis protein family (Altieri, 2010). It is essential for early brain development and neural repair, due to its protective effects on neuronal cells (Adida et al., 1998; Jiang et al., 2005). Its presence has been confirmed in the retina of various mammalian species, and it is speculated that it may play a role in modulating or preventing retinal degeneration (Downs et al., 2015) as other pro-survival genes from this family are downregulated in dogs with rod-cone degeneration (Genini et al., 2013). Thus, because the harmful effects of short-wavelength light can include retinal degeneration (Ham et al., 1976), it would be interesting to investigate whether changes in *Birc5* expression in the retina are associated with exposure to monochromatic light at various wavelengths.

Therefore, our objective was to investigate the profiles of *Rho*, *Opn4, c-Fos*, and *Birc5* mRNA expression under exposure to white and monochromatic light (blue, red, green) at 150 lx in albino rats that were maintained under a cycle of 12 h of light and 12 h of darkness. To investigate the general morphology of the outer retina, light and electron microscopy was performed.

## Materials and methods

### Animals and experimental design

Three-month-old male and female Wistar rats were used. All rats were kept in 12 h of white fluorescent light and 12 h of darkness for the first 3 months of life. White light (≤ 100 lx) was provided with fluorescent lamps.

At 3 months of age, the animals were divided into three groups that were exposed to monochromatic light (blue, green, and red-light exposure–referred to hereafter as the blue-light, green-light, and red-light groups) and one control group (*n* = 32 rats in each group, *n* = 4 from each group were sampled at each time point). Rats in the blue-light group were exposed to 12 h of blue light and 12 h of darkness for 7 day, and rats in the green- and red-light groups were exposed to their respective types of light at the same times. The control group was kept in 12 h of white fluorescent light and 12 h of darkness from birth until the end of the experiment. In all groups, photophase lasted from 20:00 to 08:00, and scotophase lasted from 08:00 to 20:00. The diagram presenting experimental design and timeline of the experiment is presented in Supplementary Figure 1.

During the experiment, all rats were kept in transparent, plexiform cages. Blue, green, and red light were provided by light emitting diode strips (blue 463 ± 10 nm; green 523 ± 10 nm; red 623 ± 10 nm). For the control group, the light was provided by fluorescent lamps that emit broad spectrum light with discrete frequency peaks. The illumination at the level of the rats’ eyes (150 lx) was measured with a monochromatic light meter (Multi-Led TENMARS, Taiwan) or a Standard ST-8820 Environmental Meter (for white light). Additionally, to determine the power of each LED light, we used a laser power meter (Power Meter Gigahertz – Optik PT 9610), which showed similar values for each kind of LED light exposure, i.e., 65–75 μW. The irradiance values for red, green, and blue light were 3.3–3.8 W/m^2^. For the light-source location and other details of the experimental system, see Supplementary Figure 2. For sample collection, rats were euthanized in a CO2 chamber every 3 h, starting at 20:00, then at 23:00, 02:00, 05:00, 08:00, 11:00, 14:00, and 17:00 (Supplementary Figure 1). From 08:00 to 17:00 samples were collected in dim red light. All experimental procedures on rats were performed in accordance with Polish and EU law (AWG FVM UWM in Olsztyn opinion for project NCN 2017/01/X/NZ4/00838, 13 October 2017).

### qPCR

For this assay, the entire retina from one eye was gently dissected form the posterior segment of the eye and placed in RNAlater™ (Invitrogen,™ United States) and stored at−80°C (for sample assignation please see Supplementary Figure 1). Total RNA was isolated from all samples using a GeneMATRIX Universal RNA Purification Kit (Eurx, Poland) following the manufacturer’s protocol. The quality and quantity of mRNA was evaluated using a NanoVue^®^ Spectrophotometer (Thermo Fisher Scientific). Reverse transcription was conducted with a RevertAid First Strand cDNA Synthesis Kit (Thermo Fisher Scientific). Each 20 μl reverse transcription reaction contained 0.5 μg of total RNA. The reverse transcription reactions were performed in a Biometra Thermocycler (Biometra T Gradient Professional Basic) for 5 min at 25°C, 60 min at 42°C, 5 min at 85°C, and finally held for 10 min at 4°C. All samples were then frozen at −80°C.

#### Gene expression examination

The reverse transcription products were diluted 1:2 with DNA- and RNA-free water. Each 20 μl PCR reaction mixture contained 1 μl of diluted RT product, 10 μl SYBR^®^ Select Master Mix (Thermo Fisher Scientific), 1 μl of primer mix (5 μM each), and 8 μl of nuclease free water. The relative expression of the four examined genes in each sample was examined in triplicate on a 96-well plate with glyceraldehyde-3-phosphate dehydrogenase (GAPDH) serving as a reference gene (the stability of GAPDH’s expression was previously tested) (primer data in Supplementary Table 1). Reactions containing no reverse transcription products served as a negative control. Reactions were incubated in a 7,500 Fast Real Time PCR System (Applied Biosystems) for 10 min at 95°C, followed by 40 cycles of 95°C for 15 s and 60°C for 1 min. After completion of qPCR, the threshold value for each gene was automatically configured above the baseline displayed in the amplification plot. The relative expression of each gene was quantified using the comparative CT method (Schmittgen and Livak, 2008).

### Light and electron microscopy

For light microscopy, retinal samples with the adjacent choroid and sclera were collected from the central region of the fundus within three minutes of heart stoppage. Samples were fixed (2 h, 4°C) in a 4% paraformaldehyde or in a mixture of 1% paraformaldehyde and 2.5% glutaraldehyde in 0.2 M phosphate buffer (pH 7.4), then were washed, postfixed in 2% osmium tetroxide (2 h at room temperature), and embedded in Epon 812. Semithin sections were stained with toluidine blue. Retinas from six rats (collected at 11:00, *n* = 4 and 14:00, *n* = 2) of each group were taken for measurements of the ONL, photoreceptor outer and inner segment thickness. Additionally, in the blue-light group, the vesicles (in the photoreceptor outer segments layer) were counted manually in a 1,300 μm long area of two semithin sections from retinas collected at 11:00 (*n* = 4) and 14:00 (*n* = 2). Counting of the vesicles and measurements of ONL and photoreceptor segments were performed with CaseViewer (3DHISTECH). Visualization of samples was performed using an AxioImager motorized light microscope and an AxioCam MRc5 digital camera (Carl Zeiss, Germany). Ultrathin sections were contrasted with uranyl acetate and lead citrate, then examined using a Tecnai 12 Spirit G2 BioTwin transmission electron microscope (FEI, United States). Five to seven cross-sections of each retina were examined by TEM.

### Statistical analyses

Before analysis, values of mRNA relative expression were log transformed because both experiments (Ganger et al., 2017) and theory (Beal, 2017) indicate that these values are log-normally distributed and thus should be transformed before statistical analysis with so-called “parametric” methods. *P*-values and 95% confidence intervals (95% CIs) for comparisons to the control group at each time of sample collection were calculated with Dunnett’s test. For accurate results, this test requires that the assumptions of normally distributed values and homogeneity of variance are met. Therefore, these assumptions were verified with normal quantile-quantile plots and plots of residuals vs. mean values, respectively. All calculations were performed with R, version 4.0.4 (R Core Team, 2021).

## Results

### Light and electron microscopy

**FIGURE 1:**
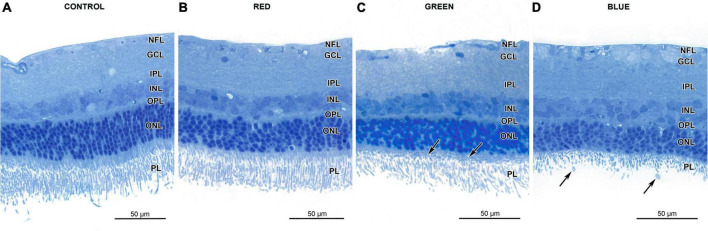
Effect of monochromatic light exposure on the retinal morphology of Wistar rats. The rats were exposed to 12 h white light (150 lx) and 12 h darkness for 7 days (control, 32 rats), 12 h red light (150 lx) and 12 h darkness for 7 days (red-light group, 32 rats), 12 h green light (150 lx) and 12 h darkness for 7 days (green-light group, 32 rats) and 12 h blue light (150 lx) and 12 h darkness for 7 days (blue-light group, 32 rats). The figure presents retinal sample collected at 11:00. Resin-embedded semithin sections were stained with toluidine blue and visualized with light microscope (AxioImager, Zeiss). Retinas from the control **(A)**, red-light **(B)** groups display normal morphology. The photoreceptor inner segments in retinas from green-light group are shorter than those of control group (arrows) **(C)**. Retinas from the blue-light group display loss and vesiculation of photoreceptor outer segments (arrows) **(D)**. Note thinner outer nuclear layer in this group.

Light microscopy did not reveal any signs of retinal damage in the control and in the red-light groups (Figures 1A,B). In the green-light group photoreceptor inner segments were disorganized and shrunken compared to the control group (Figure 1C). Loss of the photoreceptor outer segments and their vesiculation were present in rats from the blue-light group (Figure 1D). In this group, the vesicles were commonly observed in retinal samples collected at 11:00 and 14:00 and the number of those vesicles was 46–76 per rat.

**FIGURE 2:**
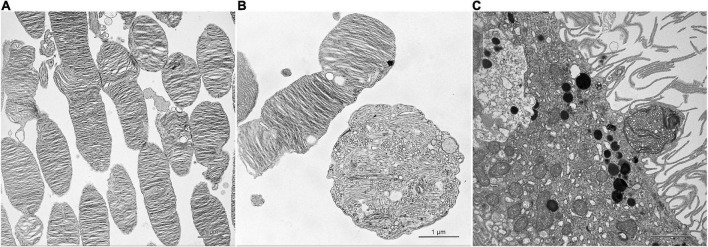
Effect of monochromatic blue light exposure on the photoreceptor outer segments and retinal pigment epithelium morphology in Wistar rats. The rats were exposed to 12 h white light (150 lx) and 12 h darkness for 7 days (control, 32 rats), and 12 h blue light (150 lx) and 12 h darkness for 7 days (blue-light group, 32 rats). The figure presents retinal sample collected at 11:00. Ultrathin sections were visualized with transmission electron microscope (Tecnai, FEI). Photoreceptor outer segments display normal morphology in the control group **(A)**. In the blue-light group, these segments form round structures with vesicles and tubules **(B)**. Retinal pigment epithelium shows long and thin microvilli and ellipsoid structure trapped between them **(C)**.

In electron micrographs, the photoreceptor outer segments looked normal in the red-light, green-light and control groups (Figure 2A). In the blue-light group, those outer segments were usually isolated from the inner segments and their apical parts formed round structures with vesicles and tubules (Figure 2B). Some of those structures were trapped between long and thin microvilli of the retinal pigment epithelium (Figure 2C). The photoreceptor inner segments appeared normal in all groups.

**FIGURE 3:**
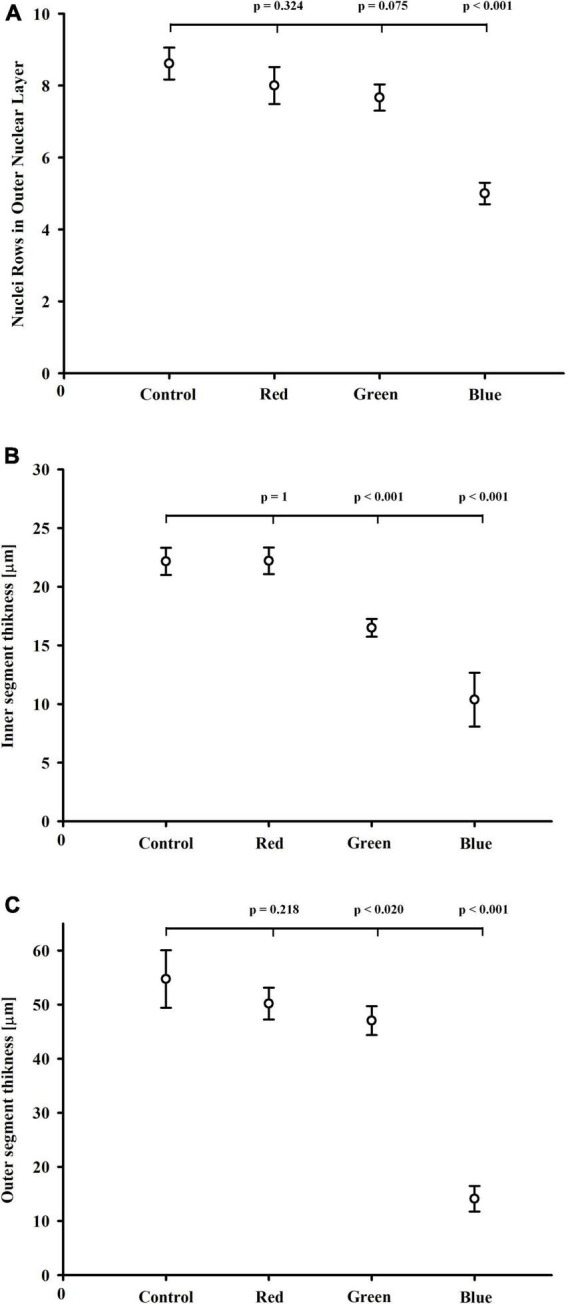
Effect of monochromatic light exposure on the outer nuclear layer and photoreceptor segment thickness. The rats were exposed to 12 h white light (150 lx) and 12 h darkness for 7 days (control, 32 rats), 12 h red light (150 lx) and 12 h darkness for 7 days (red-light group, 32 rats), 12 h green light (150 lx) and 12 h darkness for 7 days (green-light group, 32 rats) and 12 h blue light (150 lx) and 12 h darkness for 7 days (blue-light group, 32 rats). Retinal samples were collected at 11:00 (*n* = 4) and 14:00 (*n* = 2). *P*-values and 95% confidence intervals were calculated with Dunnett’s test. **(A)** Nuclei rows in the outer nuclear layer in retinas from control, red-, green-, and blue-light exposure groups. Photoreceptor inner **(B)** and outer **(C)** segment thickness in the control, red-, green-, and blue-light exposure groups.

The morphometric analysis showed, that blue-light exposure group had significantly (*p* < 0.001) fewer nuclei rows in ONL than the control group (Figure 3A). The number of nuclei rows in ONL was similar in the control, red-, and green-light exposure groups (*p* = 0.324; *p* = 0.075, respectively). The mean thickness of the photoreceptor inner segments was substantially smaller in the blue-light (*p* < 0.001), and to a lesser extent in the green light groups (*p* < 0.001) than in the control group (Figures 3B,C). The thickness of the photoreceptor outer segments was significantly smaller in the blue-light group than in the control group (*p* < 0.001). Statistical difference in the photoreceptor outer segments thickness was also observed in the green-light group (*p* < 0.02), but this difference was much smaller than in the blue-light group.

### Exposure to monochromatic blue light decreases expression of *Rho* and *Opn4* mRNA in rat retina

#### *Rho* gene expression

**FIGURE 4:**
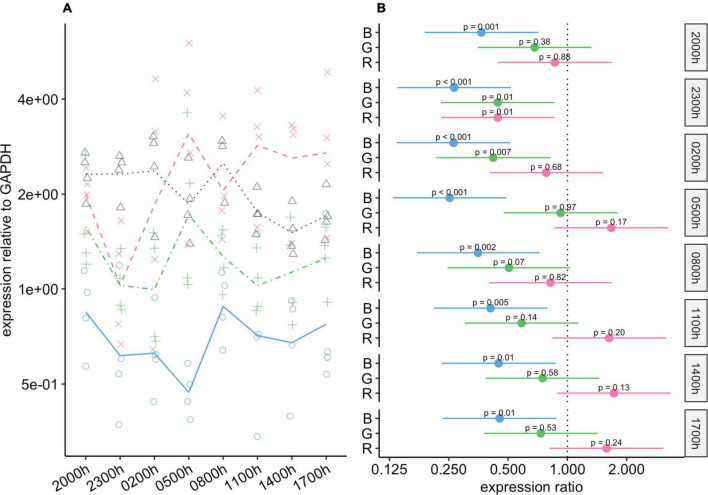
Effect of monochromatic light exposure on *Rho* mRNA expression in the retinas of Wistar rats. Experimental details were the same as in Figure 1. *P*-values and 95% confidence intervals were calculated with Dunnett’s test. Note that logarithmic scales are used. **(A)** Points show gene expression levels in individual rats; lines connect mean values in each group. To facilitate viewing by color-blind readers, different points and line types were used: control group, dotted line (••) and triangles; red-light group, dashed line (– –) and x-marks; green-light group, dot-dash line (•–) and crosses; blue-light group, solid line and circles. **(B)** Points show differences between group means; error bars show the 95% confidence interval for each difference. Abbreviations indicate which group is being compared to the control group: B, blue-light group; G, green-light group; R, red-light group.

Blue light had a substantial effect on *Rho* expression, and at all timepoints, expression of this gene was significantly lower in the blue-light group than in the control group (Figure 4). These differences were particularly noticeable from 23:00 to 05:00, when the expression of this gene was almost four-fold lower in the blue-light group, and the 95% CIs for the difference ranged from about two-fold to eight-fold lower. The mean expression of *Rho* was also lower in the green-light group than in the control group. These differences were statistically significant at 23:00 and 02:00, and close to our threshold for significance at 08:00 (*P* = 0.066). Interestingly, if it were not for one individual in the green-light group with a remarkably high value at 05:00 (Figure 4A), mean *Rho* expression would also be markedly lower in the green-light group than in the control group at this timepoint. As for the red-light group, Rho expression in this group was generally similar to that in the control group.

#### *Opn4* gene expression

**FIGURE 5:**
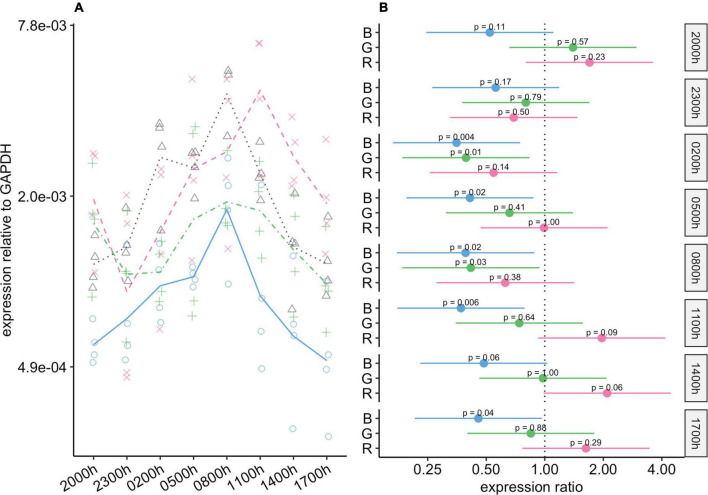
Effect of monochromatic light exposure on *Opn4* mRNA expression in the retinas of Wistar rats. Experimental details were the same as in Figure 1. *P*-values and 95% confidence intervals were calculated with Dunnett’s test. Note that logarithmic scales are used. **(A)** Points show gene expression levels in individual rats; lines connect mean values in each group. To facilitate viewing by color-blind readers, different points and line types were used: control group, dotted line (••) and triangles; red-light group, dashed line (– –) and x-marks; green-light group, dot-dash line (•–) and crosses; blue-light group, solid line and circles. **(B)** Points show differences between group means; error bars show the 95% confidence interval for each difference. Abbreviations indicate which group is being compared to the control group: B, blue-light group; G, green-light group; R, red-light group.

*Opn4* expression was affected more by blue-light exposure than by the other types of light exposure (Figure 5). At all timepoints, *Opn4* expression in the blue-light group was lower than that in the control group. At five of the timepoints, the difference was statistically significant, and at the others, although the differences were not significant, the 95% CIs for the differences included values from close to no difference to around four-fold lower in the blue-light group (Figure 5B). Compared to the control group, *Opn4* expression in the green-light group was reduced during scotophase, with statistically significant differences at 02:00 and 08:00 h. Thus, the amplitude of changes in *Opn4* levels was noticeably smaller in the green-light group than in the control group.

### Exposure to monochromatic blue light increases expression of *c-Fos* and *Birc5* mRNA in rat retina

#### *c-Fos* gene expression

**FIGURE 6:**
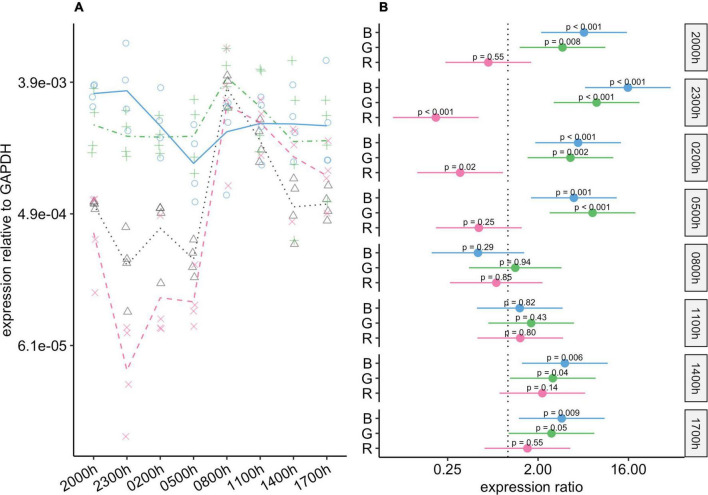
Effect of monochromatic light exposure on *c-Fos* mRNA expression in the retinas of Wistar rats. Experimental details were the same as in Figure 1. *P*-values and 95% confidence intervals were calculated with Dunnett’s test. Note that logarithmic scales are used. **(A)** Points show gene expression levels in individual rats; lines connect mean values in each group. To facilitate viewing by color-blind readers, different points and line types were used: control group, dotted line (••) and triangles; red-light group, dashed line (– –) and x-marks; green-light group, dot-dash line (•–) and crosses; blue-light group, solid line and circles. **(B)** Points show differences between group means; error bars show the 95% confidence interval for each difference. Abbreviations indicate which group is being compared to the control group: B, blue-light group; G, green-light group; R, red-light group.

In the control and the red-light groups, changes in *c-Fos* expression appeared to be rhythmic, with a peak during scotophase and a nadir during photophase (Figure 6). In the blue- and green-light groups, in contrast, rhythmic changes were markedly reduced or abolished, as *c-Fos* expression remained elevated throughout the 24-h period at levels similar to the peak levels in the red-light and control groups.

#### *Birc5* gene expression

**FIGURE 7:**
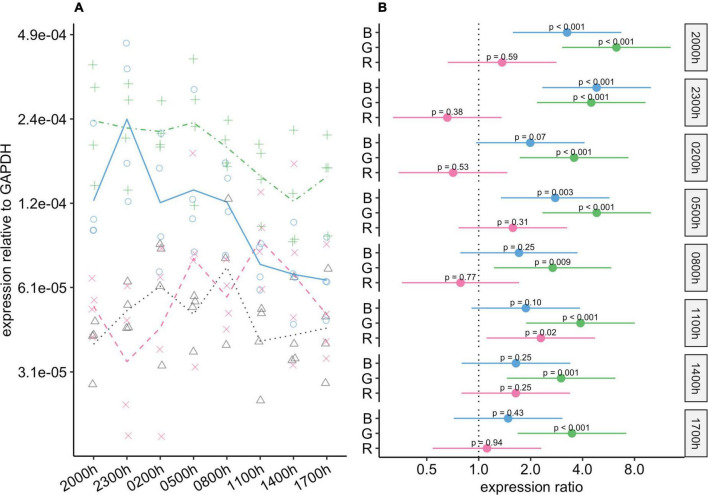
Effect of monochromatic light exposure on *Birc5* mRNA expression in the retinas of Wistar rats. Experimental details were the same as in Figure 1. *P*-values and 95% confidence intervals were calculated with Dunnett’s test. Note that logarithmic scales are used. **(A)** Points show gene expression levels in individual rats; lines connect mean values in each group. To facilitate viewing by color-blind readers, different points and line types were used: control group, dotted line (••) and triangles; red-light group, dashed line (– –) and x-marks; green-light group, dot-dash line (•–) and crosses; blue-light group, solid line and circles. **(B)** Points show differences between group means; error bars show the 95% confidence interval for each difference. Abbreviations indicate which group is being compared to the control group: B, blue-light group; G, green-light group; R, red-light group.

During photophase, expression of *Birc5* was substantially higher in the green-light and blue-light groups than in the control group, and these differences were statistically significant (Figure 7). During scotophase, *Birc5* expression continued to be markedly higher in the green-light group than in the control group. However, its expression in the blue-light group dropped during scotophase, and the 95% CIs for the differences between this group and the control group ranged from around no difference to four-fold higher in the blue-light group. *Birc5* expression in the red-light group was similar to that in the control group at all timepoints.

## Discussion

Our results indicate that exposure to blue light at 150 lx substantially reduces expression of *Opn4* and *Rho* mRNA in the retina of albino rats and increases expression of *c-Fos* and *Birc5* mRNA during photophase. They also indicate that exposure to this kind of light, but not to white or red light, causes loss and vesiculation of the photoreceptor outer segment. Also, the photoreceptor outer and inner segments are shorter in rats exposed to blue, and to a lesser extent to green light.

There are four potential reasons why monochromatic blue light could have caused changes in mRNA expression in the retina, while white light, which contains some blue light, did not. First, because blue light is only one component of white light, the intensity of blue-light exposure was less when white light was used than when monochromatic blue light was used.

Second, photoreceptors respond more strongly to blue light than to other colors of light (Grimm et al., 2001; Krigel et al., 2016; Shang et al., 2017). For example, RHO protein is sensitive to blue light at 470–480 nm (Grimm et al., 2001), and when RHO absorbs excess photons, this triggers light damage to photoreceptors (Grimm et al., 2001). Moreover, blue light exposure increases the photon catch capacity of the retina *via* photoreversal of RHO bleaching, which is much faster than metabolic regeneration of this pigment (Grimm et al., 2001). Increased photon capture means that the effects any kind of light exposure on the retina may be intensified. Thus, even though all groups were exposed to the same intensity of light, increased photon capture augmented the effects of exposure in the blue light group.

Third, blue light exposure inhibits a key mitochondrial enzyme in photoreceptors (cytochrome oxidase). This inhibition can lead to generation of oxygen species (Chen et al., 1992; Osborne et al., 2014; Núnez-Álvarez and Osborne, 2019), which can lead to inhibition of the oxidative cycle (Lawwill, 1982) and disrupt the expression of some genes in the retina, as photo-oxidative stress has been shown to be related to abnormal expression of genes related to photoreceptor survival (Chen et al., 2004).

Fourth, even though the intensity of the white light was the same as that of the blue light, the energy of the blue light was higher due to its shorter wavelength. This suggests that the damaging effects of blue light could be stronger than those of other colors of light (Grimm et al., 2001; Krigel et al., 2016; Shang et al., 2017).

As mentioned above, exposure to blue light for 7 days substantially reduced the overall levels of *Rho* in the retinas. RHO serves as a mediator of light-induced retinal degeneration (Grimm et al., 2000a,^2001^), and the amount of RHO available during light exposure determines the severity of the damage that rods suffer (Grimm et al., 2001). We speculate that the decrease in *Rho* expression that we observed, if accompanied by a decrease in RHO expression, may have prevented further light-induced rod damage. Thus, it would be interesting for future studies to investigate the expression of RHO in the rat retina under these conditions. Because the retina of albino rats is more susceptible to light damage than the retina of pigmented rats (Krigel et al., 2016), it would be also interesting for future studies to investigate changes in *Rho* expression in pigmented rats under the same conditions.

The reduction in *Rho* expression after blue light exposure may have been responsible for several other changes that we observed, as *Rho* gene expression determines rod outer segment morphology (Makino et al., 2012; Price et al., 2012). Lower *Rho* expression probably led to lower levels of RHO, which could have contributed to the moderate alterations we observed in the retinal photoreceptors, such as decreased in outer segment thickness and their vesiculation. Electron microscopy revealed that this vesiculation was characterized by round tubular structures packed in swollen outer segments. This observation is similar to that reported by Kuwabara and Gorn (1968) in rats continuously exposed to high intensity white light for several weeks. Those authors suggested that this kind of vesiculation indicates an intermediate stage of light induced retinal damage.

Our results indicate that low-intensity green light exposure also reduces *Rho* expression, but to a lesser extent than blue light exposure, and that red light exposure causes little or no change in *Rho* expression compared to white light exposure. This is most likely due to the longer wavelengths of green and red light, which mean that they are less energetic than blue light. Finally, the photoreceptor outer segments in the retinas exposed to green light were much less damaged, unlike those exposed to blue light, which may also have influenced *Rho* expression.

We showed that blue light and (to a lesser extent) green light decrease expression of *Opn4* in the rats’ retinas. This is in accordance with reports that exposure to high-intensity monochromatic blue light (1,000 lx) and cool white light (3,000 lx) reduce *OPn4* expression in ipRGCs in albino and pigmented rats, respectively (García-Ayuso et al., 2017; Ziółkowska et al., 2022). In our animals exposed to blue or green light, expression of *Opn4* mRNA peaked at the beginning of scotophase. The timing of this peak is consistent with the peaks in *Opn4* expression in the retinas of albino and pigmented rats exposed to white light during a light-dark cycle (Hannibal et al., 2005, 2013).

It is not clear whether *OPn4* mediates the damaging effect of light exposure on ipRGCs (Ziółkowska et al., 2022). It has been demonstrated that mitochondrial enzymes, which are abundant in ipRGCs, are affected by blue light, which can lead to generation of reactive oxygen species and affect axonal transport and the survival of the ipRGCs (Chen et al., 1992; Osborne et al., 2014). *OPn4* also produces reactive oxygen species when it is activated by blue light. Thus, our results and those of other researchers suggest that downregulation of *Opn4* and *OPn4* expression in response to blue-light exposure could serve as protective mechanisms.

Our observation that, in the retinas of rats kept under a light-dark cycle with white- or red-light exposure, *c-Fos* expression is highest around the beginning of scotophase and lowest during photophase is consistent with result from studies of mice (Nir and Agarwal, 1993) and rats (Yoshida et al., 1993). Taken together, this observation and those of previous researchers show that *c-Fos* is expressed in rhythmic manner in mouse and rat retinas, which suggests that it plays an important role in retinal physiology (Nir and Agarwal, 1993; Yoshida et al., 1993).

Our results show that exposure to blue or green light at an intensity of 150 lx substantially increases *c-Fos* expression during photophase, and they suggest that exposure to these kinds of light at this intensity markedly reduces or abolishes rhythmic expression of this mRNA. *c-Fos* is a proto-oncogene that Hafezi et al. (1997) found is essential for light-induced apoptosis of photoreceptors, and Oshitari et al. (2002) found that it is involved in RGCs death and regeneration. Grimm et al. (2000b) observed that, in rats exposed to pulses of low intensity (non-damaging) white light, *c-Fos* levels declined rapidly after initial peaks, but that its levels remained elevated after exposure to high intensity (damaging) light. These findings suggest that the increase in *c-Fos* expression in our rats’ retinas may have contributed to the vesiculation of the photoreceptor outer segments in these animals, which is characteristic of the early stage of reversible, moderate light damage and is similar to the alterations described in previous studies on white light exposure (Szczesny et al., 1995; Grimm et al., 2001). Additionally, (Bussolino et al., 1998, 2001) showed that *c-Fos* that is associated with the endoplasmic reticulum activates lipid synthesizing enzymes in photoreceptors and RGCs in chickens. This suggests that, if *c-Fos* levels are increased, the pool of lipids in retinal cells could be increased. These lipids could undergo peroxidation, damaging the retina, as blue light inhibits a key mitochondrial enzyme present in photoreceptors (cytochrome oxidase), which can lead to generation of oxygen species and oxidative stress in the retina (Grimm et al., 2001; Krigel et al., 2016; Shang et al., 2017).

We found that exposure to blue or green light markedly increases expression of *Birc5* in rat retinas. As mentioned above, blue light exposure at 150 lx was associated with moderate changes in the photoreceptor outer segment, which was the only suggestion of retinal damage. We speculate that damage to the mitochondria, which was observed in RGCs after rats were exposed to blue light at 1,000 lx (Ziółkowska et al., 2022), may have been prevented in part by the activity of *Birc5*. *Birc5* has anti-apoptotic effects, which may be due to a subcellular mitochondrial pool of this transcription factor that inhibits caspase-9 activation (Dohi et al., 2004). Thus, it is possible that *Birc5* expression is up-regulated to protect retinal neurons from RHO-mediated light-induced damage and that this happens before the mitochondria in retinal neurons are altered. It is also possible that exposure to high-energy blue light induced other protective mechanisms that reduced or prevented retinal damage. For example, Chen et al. (2004) found that exposure of mice retinas to high-intensity cool white LED light upregulates the expression of another anti-apoptotic gene, Bag3 (related to Bcl2 protein), which may affect the survival of photoreceptors subjected to photo-oxidative stress (Grimm et al., 2001; Chen et al., 2004).

In conclusion, our results indicate that exposure to blue light and, to a lesser degree, exposure to green light decrease expression of *Rho* and *Opn4* mRNA and increase expression of *Birc5* and *c-Fos* mRNA. Increased expression of the anti-apoptotic gene *Birc5* and decreased expression of *Rho* and *Opn4* after exposure to these kinds of light are likely to be early responses that help to reduce light-induced retinal damage. It would be interesting for future studies to investigate whether rods and cones are equally affected under these conditions and whether these kinds of light affect mitochondria.

## Data availability statement

The original contributions presented in this study are included in the article/Supplementary material, further inquiries can be directed to the corresponding author.

## Ethics statement

All experimental procedures on rats were performed in accordance with Polish and EU law concerning animal welfare (AWG FVM UWM in Olsztyn opinion for project NCN 117 2017/01/X/NZ4/00838, 13 October 2017).

## Author contributions

NZ: conception, design of the study and writing the first draft of the manuscript. NZ and BL: data analysis. BL: project administration and reviewing the manuscript. Both authors contributed to the article and approved the submitted version.

## Supplementary material

Supplementary Figure 1 Diagram of the experimental protocol including lighting conditions and sampling times. The rats were exposed to 12 h white light (150 lx) and 12 h darkness for 7 days (control, 32 rats), and 12 h of monochromatic light (blue, green, red at 150 lx) and 12 h darkness for 7 days (32 rats per group). *At 11:00 the right eye from each of four rats was collected for qPCR and the left eye for transmission electron microscopy (TEM). *At 14:00 the right eye from 2 rats was collected for TEM, and the left eye from those two rats for qPCR. The eyes from two other rats at this timepoint were collected for qPCR only.

Supplementary Figure 2 Enclosure for experimental light exposure. Blue, green, and red light were provided by light emitting diode strips (blue 463 ± 10 nm; green 523 ± 10 nm; red 623 ± 10 nm).
