# Supplementary figures and images for: Profiles of Rho, Opn4, c-Fos, and Birc5 mRNA expression in Wistar rat retinas exposed to white or monochromatic light
Source: Front Neuroanat. 2022 Aug 18;16:956000. doi: 10.3389/fnana.2022.956000 (PMC9434339; doi:10.3389/fnana.2022.956000)

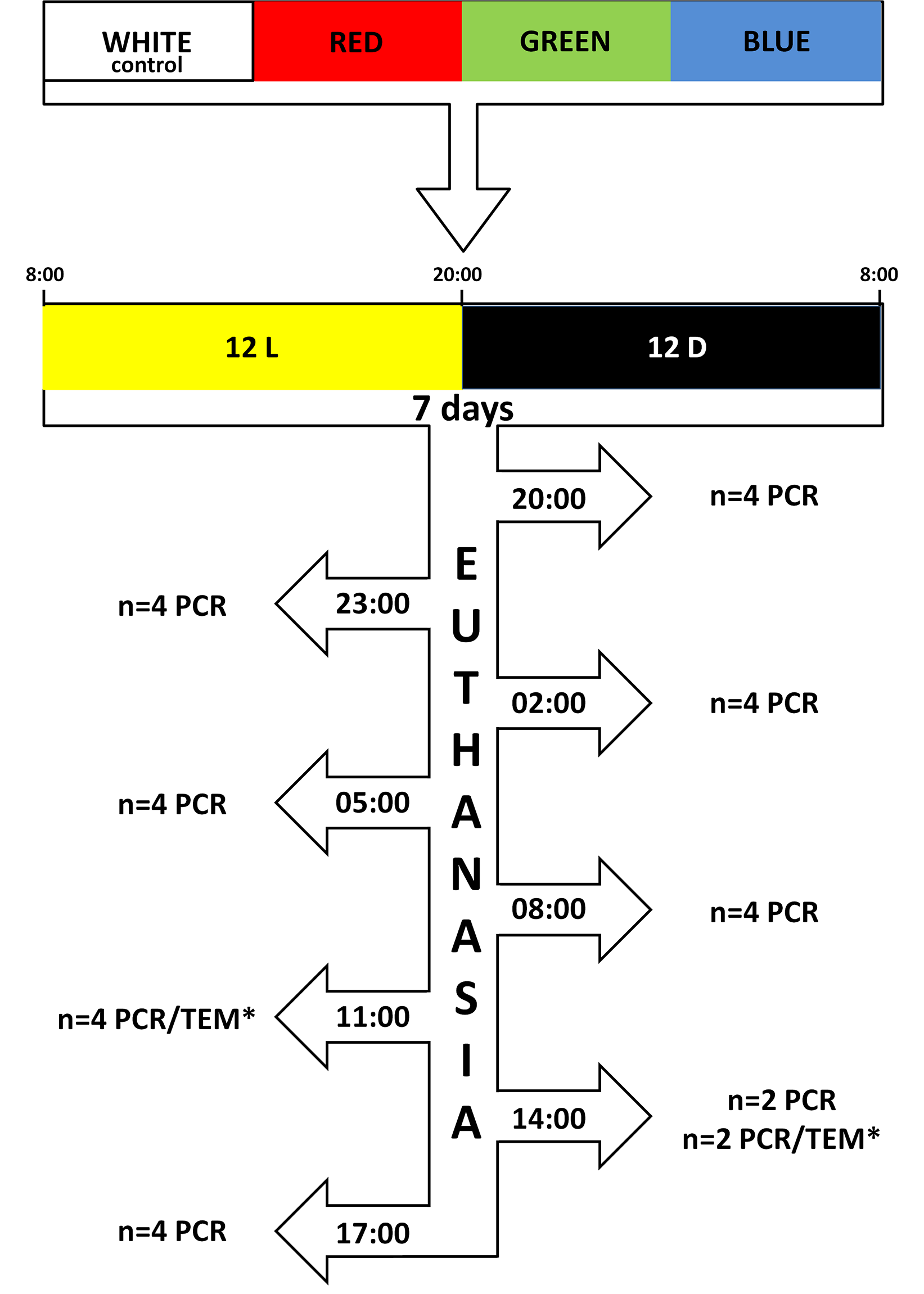

Supplement: Supplementary Figure 1 — Diagram of the experimental protocol including lighting conditions and sampling times. The rats were exposed to 12 h white light (150 lx) and 12 h darkness for 7 days (control, 32 rats), and 12 h of monochromatic light (blue, green, red at 150 lx) and 12 h darkness for 7 days (32 rats per group). *At 11:00 the right eye from each of four rats was collected for qPCR and the left eye for transmission electron microscopy (TEM). *At 14:00 the right eye from 2 rats was collected for TEM, and the left eye from those two rats for qPCR. The eyes from two other rats at this timepoint were collected for qPCR only. [file Image_1.TIF]

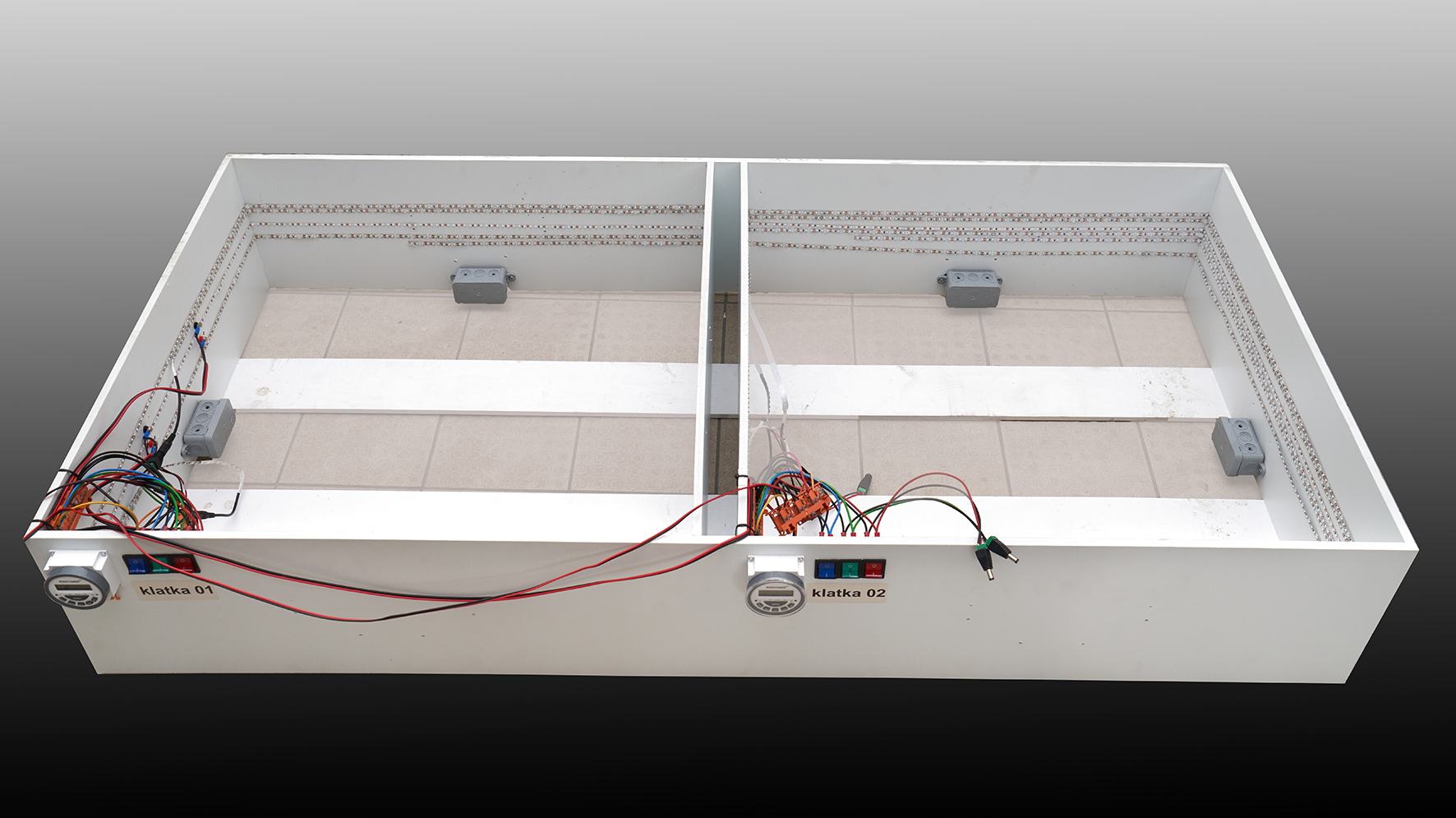

Supplement: Supplementary Figure 2 — Enclosure for experimental light exposure. Blue, green, and red light were provided by light emitting diode strips (blue 463 ± 10 nm; green 523 ± 10 nm; red 623 ± 10 nm). [file Image_2.TIF]
