# Supplementary material for: Profiles of Rho, Opn4, c-Fos, and Birc5 mRNA expression in Wistar rat retinas exposed to white or monochromatic light
Source: Front Neuroanat. 2022 Aug 18;16:956000. doi: 10.3389/fnana.2022.956000 (PMC9434339; doi:10.3389/fnana.2022.956000)
Supplement: Supplementary file 3 [file Table_1.DOCX]

Supplementary Table 1. Primer data for qPCR analysis.

| **Gene name** |  |  | 5’ 3’ | **Exon junction** | **Product length (bp)** |
| --- | --- | --- | --- | --- | --- |
| glyceraldehyde-3-phosphate dehydrogenase (Gapdh) | NM_017008.4 | Forward | CATGGCCTTCCGTGTTCCTA |  | 74 |
|  |  | Revers | ACTTGGCAGGTTTCTCCAGG | 825/826 |  |
| baculoviral IAP repeat-containing 5 (Birc5) | NM_022274.1 | Forward | GGATGACAACCCTATAGAGGAGC | 225/226 | 89 |
|  |  | Revers | TGACGGTCAGTTCTTCCACC |  |  |
| Opsin 4 (Opn4) | NM_138860.1 | Forward | CTGAGAGTGAAGTGGGCTGG | 1443/1444 | 125 |
|  |  | Revers | GCTGGAAGGAGCCTTGACTT |  |  |
| Fos proto-oncogene, AP-1 transcription factor subunit (c-Fos) | NM_022197.2 | Forward | AGATACGCTCCAAGCGGAGA | 653/654 | 141 |
|  |  | Revers | TCATTGGGGATCTTGCAGGC |  |  |
| rhodopsin (Rho) | NM_033441.1 | Forward | CCACCCTTGGAGGTGAAATCG | 444/445 | 104 |
|  |  | Revers | ATTCTCCCCAAAGCGGAAGT |  |  |
